# Epitype-inducing temperatures drive DNA methylation changes during somatic embryogenesis in the long-lived gymnosperm Norway spruce

**DOI:** 10.3389/fpls.2023.1196806

**Published:** 2023-07-20

**Authors:** Marcos Viejo, Torstein Tengs, Igor Yakovlev, Hugh Cross, Paal Krokene, Jorunn E. Olsen, Carl Gunnar Fossdal

**Affiliations:** ^1^ Department of Molecular Plant Biology, Norwegian Institute of Bioeconomy Research, Ås, Norway; ^2^ Department of Plant Sciences, Faculty of Biosciences, Norwegian University of Life Sciences, Ås, Norway; ^3^ Department of Functional Biology, University of Santiago de Compostela, Santiago de Compostela, Spain; ^4^ Department of Breeding and Genetics, Norwegian Institute of Food, Fisheries and Aquaculture Research (NOFIMA), Ås, Norway; ^5^ Department of Science, National Ecological Observatory Network, Boulder, CO, United States

**Keywords:** embryogenesis, DNA methylation, epigenetic memory, epigenetic machinery, spruce

## Abstract

An epigenetic memory of the temperature sum experienced during embryogenesis is part of the climatic adaptation strategy of the long-lived gymnosperm Norway spruce. This memory has a lasting effect on the timing of bud phenology and frost tolerance in the resulting epitype trees. The epigenetic memory is well characterized phenotypically and at the transcriptome level, but to what extent DNA methylation changes are involved have not previously been determined. To address this, we analyzed somatic epitype embryos of Norway spruce clones produced at contrasting epitype-inducing conditions (18 and 28°C). We screened for differential DNA methylation in 2744 genes related mainly to the epigenetic machinery, circadian clock, and phenology. Of these genes, 68% displayed differential DNA methylation patterns between contrasting epitype embryos in at least one methylation context (CpG, CHG, CHH). Several genes related to the epigenetic machinery (e.g., DNA methyltransferases, *ARGONAUTE*) and the control of bud phenology (*FTL* genes) were differentially methylated. This indicates that the epitype-inducing temperature conditions induce an epigenetic memory involving specific DNA methylation changes in Norway spruce.

## 1 Introduction

Global warming challenges an organism’s ability to adapt quickly enough to rapid temperature change. In classical quantitative genetics, phenotypic variation is considered to result from the genotype, environmental variation, and their interactions. However, also epigenetic changes contribute to environmental variance increasing the phenotypic variation (Hirsch et al., 2012; Le Gac et al., 2018). An intriguing biological question is whether molecular mechanisms that induce epigenetic memory in response to environmental conditions can generate better adapted offspring. Such a molecular memory allow e.g., plants to adapt more rapidly to global warming (Bräutigam et al., 2013).

Plants synchronize their annual growth cycle with environmental conditions to maximize their performance during the growing season. This is especially important at high latitudes, where annual temperature oscillations and photoperiod regimes range from moderate to extreme. In Europe, the conifer Norway spruce (*Picea abies*) has formed clines that reflect crucial adaptive traits to local conditions (Ekberg et al., 1979). This clinal variation has developed despite the species’ evolutionary recent recolonization of vast areas following the last ice age. Key adaptive traits in this species are (i) the timing of bud burst during spring to avoid late spring frosts and (ii) timing of bud set to achieve the required level of frost hardening before winter (Skrøppa et al., 2007). These adaptive traits, previously thought to be under strict Mendelian inheritance, are also adjusted during embryogenesis depending on the temperature sum (Skrøppa et al., 2010). Strikingly, individuals have so far been shown to retain this phenological memory for more than 20 years, suggesting a life-lasting epigenetic change (Skrøppa et al., 2007; Carneros et al., 2017). Johnsen et al. (2005) proposed that this adjustment of key phenological traits is laid down as an epigenetic memory during embryogenesis and seed development.

In a ground-breaking experiment, (Kvaalen and Johnsen, 2008) firmly established that contrasting temperature sums induce the same epigenetic memory effect during *in vitro* somatic embryogenesis (asexual clonal propagation) as observed during zygotic embryogenesis (sexual propagation) in Norway spruce. Thus, the temperature conditions experienced during embryogenesis are a driving force for epigenetic memory independent of maternal influences. Using genetically identical somatic embryos, different temperature sums, called epitype-inducing temperatures (or epi-temperatures) were shown to affect bud phenology and gene expression in the resulting plants in a predictable and reproducible manner (Yakovlev et al., 2016; Carneros et al., 2017). Plants grown from clonal embryos developing at different epi-temperatures are genetically identical but phenotypically different under common garden conditions and are known as epitypes (Yakovlev et al., 2012). The epigenetic nature of this memory is substantiated by the observation that distinctive small RNA populations, including miRNA in epitype embryos, are differentially expressed and appear to be involved in the regulation of epigenetic machinery-related gene expression (Yakovlev and Fossdal, 2017). As in sexual reproduction (Skrøppa et al., 2007), the epigenetic memory formed during asexual reproduction by somatic embryogenesis in Norway spruce is long-lasting. Even after nearly two decades in a common garden, bud burst timing remains significantly shifted between epitypes, demonstrating that the memory is stable and maintained through mitotic propagation through shifting seasons, year after year (Carneros et al., 2017).

Clonal Norway spruce epitypes have distinct transcriptomic profiles for genes involved in the epigenetic machinery (Yakovlev et al., 2016; Carneros et al., 2017). Carneros and coworkers also found a shift in expression of specific genes related to de-hardening (e.g., dehydrins) and bud dormancy (*FLOWERING LOCUS T-TERMINAL FLOWER-1-LIKE 2* gene; *PaFTL2*) in buds and needles of different epitype trees over the bud burst period. A similar stress memory that modulates gene expression and affects plant performance has been described previously in angiosperm plants such as *Arabidopis thaliana*. This includes priming mechanisms resulting in a short-term memory that fades away when the stressor disappears (Chinnusamy and Zhu, 2009a). However, in long-lived plants such as conifers the epigenetic mechanisms underpinning long-lasting temperature-induced memory have remained elusive due to the extreme complexity of conifer genomes and the very long generation times of such trees (Seymour and Becker, 2017).

DNA methylation, which is the most studied epigenetic mark in plants, plays an essential role both in normal development and as an intermediate in the transduction of external stimuli leading to changes in gene expression (Chinnusamy and Zhu, 2009b). During embryogenesis, strict control of DNA methylation is crucial in order to correctly trigger polarity acquisition, embryo shape, meristem organization, and cell identity (Xiao et al., 2006). In addition, tissue-dependent dynamic changes in DNA methylation at the sequence level have been described in the *A. thaliana* genome during zygotic embryogenesis (Kawakatsu et al., 2017). However, *A. thaliana* has a short life span, small genome (135 Mb), low DNA methylation levels, low content of transposable elements (Alonso et al., 2015), and lack of overwintering buds. It is therefore not a suitable model for studying epigenetic memory in very long-lived plants such as the gymnosperm Norway spruce with its massive 19.6 Gb, highly methylated genome rich in transposable elements. The sequencing of the Norway spruce genome by Nystedt et al. (2013) was a milestone, together with the first Norway spruce methylome published by Ausin et al. (2016), which also provided the first reference for single nucleotide-level cytosine methylation in embryonic materials in this species.

The most well know epigenetic mechanism related to temperature is vernalization, the memory of cold temperatures (winter) that induces some plants to flower. The vernalization mechanism is very well described in winter annual ecotypes of the model angiosperm *A. thaliana*, where this mechanism is reset in each generation (Tao et al., 2017). Still, the long-lasting epigenetic memory of temperature during embryogenesis that affects the timing of the yearly phenological events in the long-lived gymnosperm Norway spruce (Carneros et al., 2017) is likely to be significantly different from the vernalization mechanism. Additionally, to our knowledge there are no previous reports for any plant species on how temperature conditions during embryogenesis induce differential DNA methylation patterns and result in the establishment of an epigenetic memory.

To explore the molecular basis of the formation of the epigenetic memory in Norway spruce we examined epitype embryos for differences in their DNA-methylome and transcriptome. We characterized DNA methylation patterns in shoot and root apical meristem-containing parts of mature somatic embryos generated at two contrasting epi-temperatures. Due to the massive size of the Norway spruce genome, we used a targeted approach to address the DNA methylation status at a single-base resolution in the promotor and start of specific target genes, including genes involved in the timing of phenology, the epigenetic machinery, and the circadian clock. Because the epigenetic memory in Norway spruce is known to be induced during embryogenesis, the memory must involve altered expression of epigenetic machinery-related genes involved in creating or erasing epigenetic marks. We analyzed the transcriptomes in immature and mature embryos to pinpoint which parts of the epigenetic machinery that are involved in shaping differential methylation patterns under epitype-inducing temperature conditions.

## 2 Material and methods

### 2.1 Plant materials

Clonal somatic embryos were generated by resuming the growth of cryopreserved proliferative calli induced from a single Norway spruce clone (B10V). This clone is known to develop into epitypes with large difference in bud phenology, depending on the epi-temperature experienced during somatic embryo development (Carneros et al., 2017). Initially, embryogenic calli were induced from a zygotic embryo in a seed from a controlled cross described by Kvaalen and Johnsen (2008). The somatic embryos (epitypes) were generated according to Yakovlev et al. (2014) at two contrasting epi-temperatures: 18°C (cool) and 28°C (warm). Mature and immature cotyledonary embryos were harvested from both epitypes after 4 and 3 months of culturing, respectively, frozen in liquid nitrogen, and stored at -80°C until further analyses. Immature embryos were smaller than mature embryos and their cotyledons were not completely developed and less yellow in color, indicating that they were more hydrated than the mature embryos.

The frozen embryos were dissected into an apical and basal part before they were further processed for targeted bisulfite sequencing and RNA-Sequencing (RNA-Seq). The apical embryo part comprised the cotyledons and shoot apical meristem (SAM), whereas the basal part comprised the hypocotyl and root apical meristem (RAM). For analysis of DNA methylation, we used three biological replicates consisting of pools of 5 apical or 5 basal embryo parts of mature embryos per epitype (a total of 3 x 2 x 2 = 12 samples). For RNA-sequencing (RNA seq), we used corresponding samples of both immature and mature embryos (a total of 3 x 2 x 2 x 2 = 24 samples).

### 2.2 Targeted bisulfite sequencing and selection of genes

Total DNA extraction of 12 samples of mature embryos was performed using the DNeasy Plant Mini Kit (Qiagen Sciences Inc, Germantown, MD, USA, cat. no. 69104) following the manufacturer´s protocol. DNA quality and concentration were assessed using a Nanodrop 2000 spectrophotometer (Thermo Scientific, Waltham, MA, US). After shearing DNA using a M220 Focused-ultrasonicator (Covaris, Inc., Woburn, MA, US), DNA fragments were bisulfite-converted using the EZ DNA Methylation-Gold Kit (Zymo ResearchCorp, Orange, CA, USA). Libraries were made using the SeqCap Epi Enrichment system (Roche, Basel, Switzerland), following the manufacturer´s instructions (see further description of this system below).

To achieve a broad screening of cytosine methylation levels, we analyzed a comparable 3 kb region of a total of 2744 genes. We focused on high confidence-genes and genes previously shown to be differentially expressed in epitype trees or embryos that had developed under the two different temperatures, including genes previously described to be involved in epigenetic or phenology regulation (Yakovlev et al., 2016; Carneros et al., 2017). Capture probes targeting 2 kb of the promoter and 1 kb of the coding sequence (exon) upstream and downstream from the start codon, respectively, were designed for each gene region by Roche using the custom SeqCap Epi technology. Using the coding sequence, all target gene sequences were annotated using the software Blast2GO (Conesa and Götz, 2008). The analysis was done using the ‘viridiplantae’-subset of the non-redundant database (with all other parameters set to default). Of the 2744 target gene regions, 2479 (90.3%) were successfully annotated (**Supplementary Table 1**) and assigned to 18 functional categories: epigenetic, chromatin components, growth and development, response to stress, transcription factors, cell cycle, kinases, hormone metabolism, chloroplast, mitochondria, organelles and cell traffic, membrane components, cell wall, protein anabolism, protein catabolism, enzymes, carbohydrate metabolism, and lipid metabolism. The remaining 265 genes did not correspond with a known function and were denoted as ‘unknown’. These were included in the analysis since they were high-confidence gene models previously shown to be differentially expressed in epitype-embryos (Yakovlev et al., 2016). Genomic sequence information for each gene was extracted from version 1.0 of the Norway spruce genome (www.http://congenie.org/).

Targeted genes were sequenced at the Norwegian Sequencing Center (Oslo, Norway), using a HiSeq 3000 sequencing machine (Illumina) with 150 basepair (bp), paired-end reads. Sequences were trimmed using the software trimmomatic (version 0.36) (Bolger et al., 2014), with leading and trailing low-quality trimming set to three nucleotide positions, minimum sequence length set to 36 nucleotides, and sliding window setting ‘4:15’. Cytosine methylation levels were estimated using the software Bismark (version 0.17.0) (Krueger and Andrews, 2011), where trimmed sequences were non-directionally aligned to target sequences using the bowtie1 algorithm. The fraction of reads showing C to U conversions was calculated for all the interrogated cytosine positions in the genomic contexts CpG (cytosine preceding guanine), CHG, and CHH (where H corresponds to A, T or C).

### 2.3 Calculation of percentages of methylated and unmethylated cytosines

For each cytosine position in the target sequences (and their reverse complements), the fraction of reads reported by Bismark as methylated was calculated and expressed as percent methylation (number of reads with methylated C/total number of reads). The average methylation percentage for a given gene was calculated by counting the total interrogated number of methylated and unmethylated cytosines along the gene’s sequence using the strategy described above (number of methylated C’s/total number of C’s, averaged over all reads). The average methylation level for each DNA methylation context (CpG, CHG and CHH) was calculated by dividing the total number of cytosines counted as methylated by the multiplication of the total cytosines counted in that context type and the number of contexts along the sequence of each target gene [number of methylated C’s in context X/(total number of C’s in context X × number of contexts in gene)].

### 2.4 Identification of differentially methylated regions and genes

Each target gene was split into 60 consecutive 50-bp windows to obtain readable plots of cytosine methylation. For each window, the number of methylated and non-methylated cytosines for the two epitypes was counted, and two-tailed Fisher’s exact tests were performed on the 2 × 2 contingency tables. A gene was identified as differentially methylated when the difference in methylation level counts between the two epitypes in one or more 50 bp region was significant (p-value < 0.05) and the difference in methylation level was > 20%. Each such 50 bp region was considered a differentially methylated region (DMR).

### 2.5 RNA sequencing

We did RNA-Seq of the apical and basal part of another set of epitypes generated at cool and warm temperatures, using both immature and mature embryos. Both developmental stages were treated and sampled as described above (‘Plant materials’): three biological replicates, each consisting of five pooled embryos, were dissected into apical and basal parts. Total RNA was isolated from 24 samples using Epicenter MasterPure Plant RNA Purification Kit (Epicenter, Madison, WI, USA, #MPR09100) following the manufacturer´s instructions. RNA quantity and quality were measured using a NanoDrop 2000 spectrophotometer (Thermo Scientific, Waltham, MA, US). RNA was stored at -80°C until cDNA libraries were constructed and sequenced at the Norwegian Sequencing Center using a HiSeq 3000 sequencing machine (Illumina, Inc., San Diego, CA, USA) with 150 bp, paired-end reads.

Raw sequence reads were trimmed and filtered as described above and mapped to version 1.0 of the spruce transcriptome (http://congenie.org/) using the bwa package (Li and Durbin, 2009). The fraction of reads that could be mapped to the spruce transcriptome ranged from 72% to 77% (total number of reads from 10,133,613 to 18,174,704). Mapped reads per gene were counted using the rsem software (Li and Dewey, 2014) and differentially expressed genes (DEGs) were identified using the R wrapper SARTools (version 1.7.4) (Varet et al., 2016). Alpha was adjusted to 0.05 and other parameters were set according to the SARTools template script provided for edgeR-based analyses (https://github.com/PF2-pasteur-fr/SARTools).

## 3 Results

### 3.1 DNA methylation patterns and levels differ between epitypes and apical/basal embryo parts

**Figure 1:**
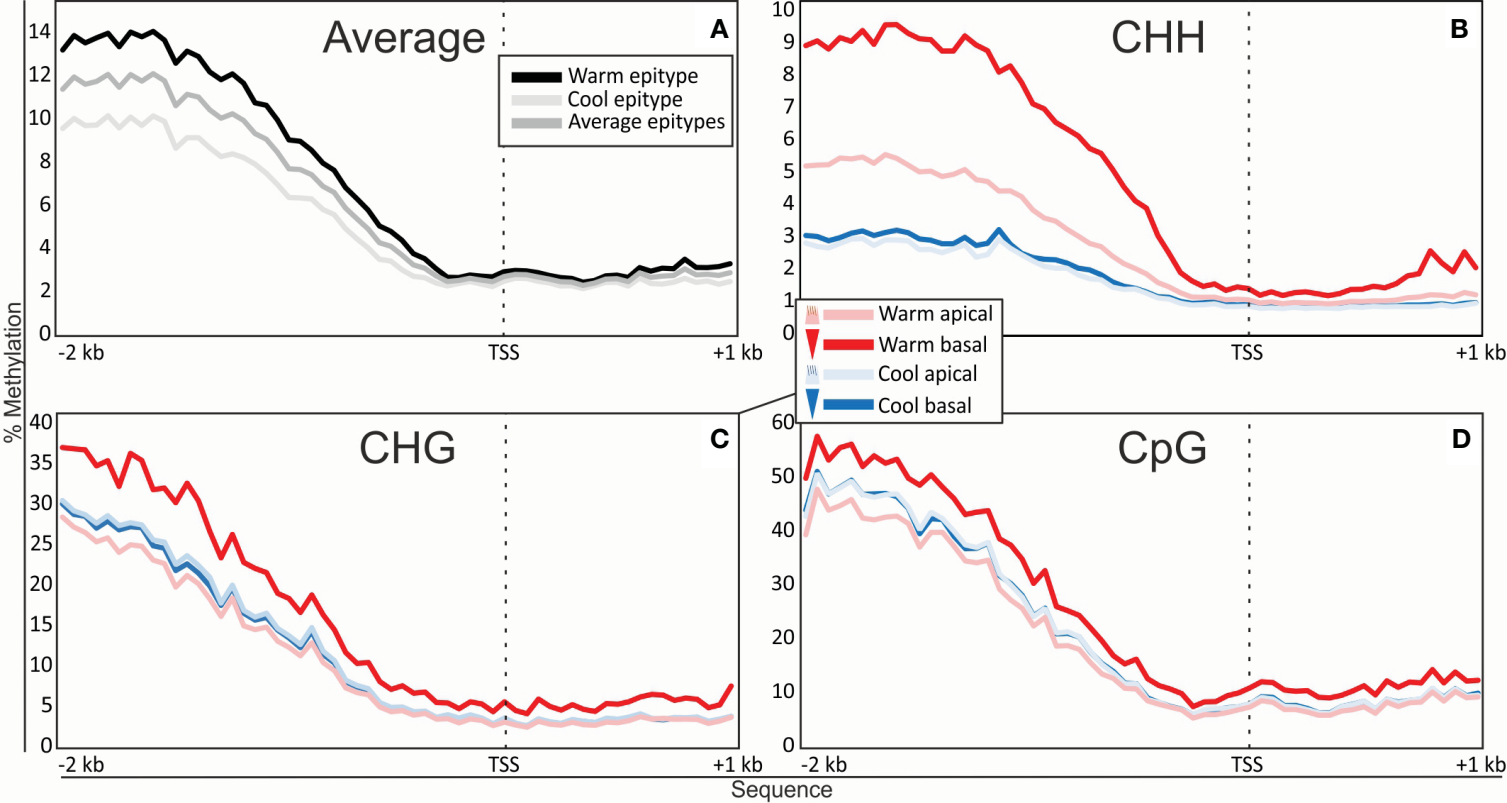
Average DNA methylation percentages in 50 bp windows along 2 kb of the promoter and 1 kb of the gene body spanning the transcriptional start site (TSS; dotted vertical line) of 2744 targeted genes in clonal mature somatic epitype embryos of Norway spruce generated at warm (28°C; red) or cool temperatures (18°C; blue). **(A)** Global methylation level per epitype (combined apical and basal embryo part). **(B–D)** Methylation level per epitype and embryo part (apical or basal) for each DNA methylation context (CHH, CHG and CpG; H corresponds to A, T or C). Data are means of three biological replicates per embryo part and epitype, with each replicate consisting of pooled materials from five embryos.

Analyses of DNA methylation levels in the apical and basal part of two different somatic embryo epitypes revealed a clear relationship between the temperature experienced during embryogenesis and the DNA methylation levels. Along the analyzed 2 kb promoter region, embryos exposed to 28°C during embryogenesis (warm epitype) showed hypermethylation relative to embryos that developed at 18°C (cool epitype) (**Figure 1A**). The average level of global DNA methylation (methylation level for all methylation contexts combined) for both epitypes varied between 2 and 12% across the analyzed 3 kb sequences. The methylation level and the difference between the epitypes were substantially lower in the first 1 kb of the gene body than in the promoter. For all three DNA methylation contexts (CHH, CHG and CpG) there were high methylation levels at the beginning of the 2 kb promoter sequence, decreasing levels towards the transcriptional start site (TSS), and low levels in the gene body (**Figures 1B–D**). The range of methylation levels per 50 bp window varied from 5-58% for CpG to 1-9.5% for CHH, while CHG had intermediated levels (2.5-36%). In the warm epitype, the basal embryo part was strongly hypermethylated relative to the apical part, while there was no clear difference between embryo parts in the cool epitype (**Figures 1B–D**).

### 3.2 Distribution of CHH, CHG and CpG sites along the 3 kb target sequences

**Figure 2:**
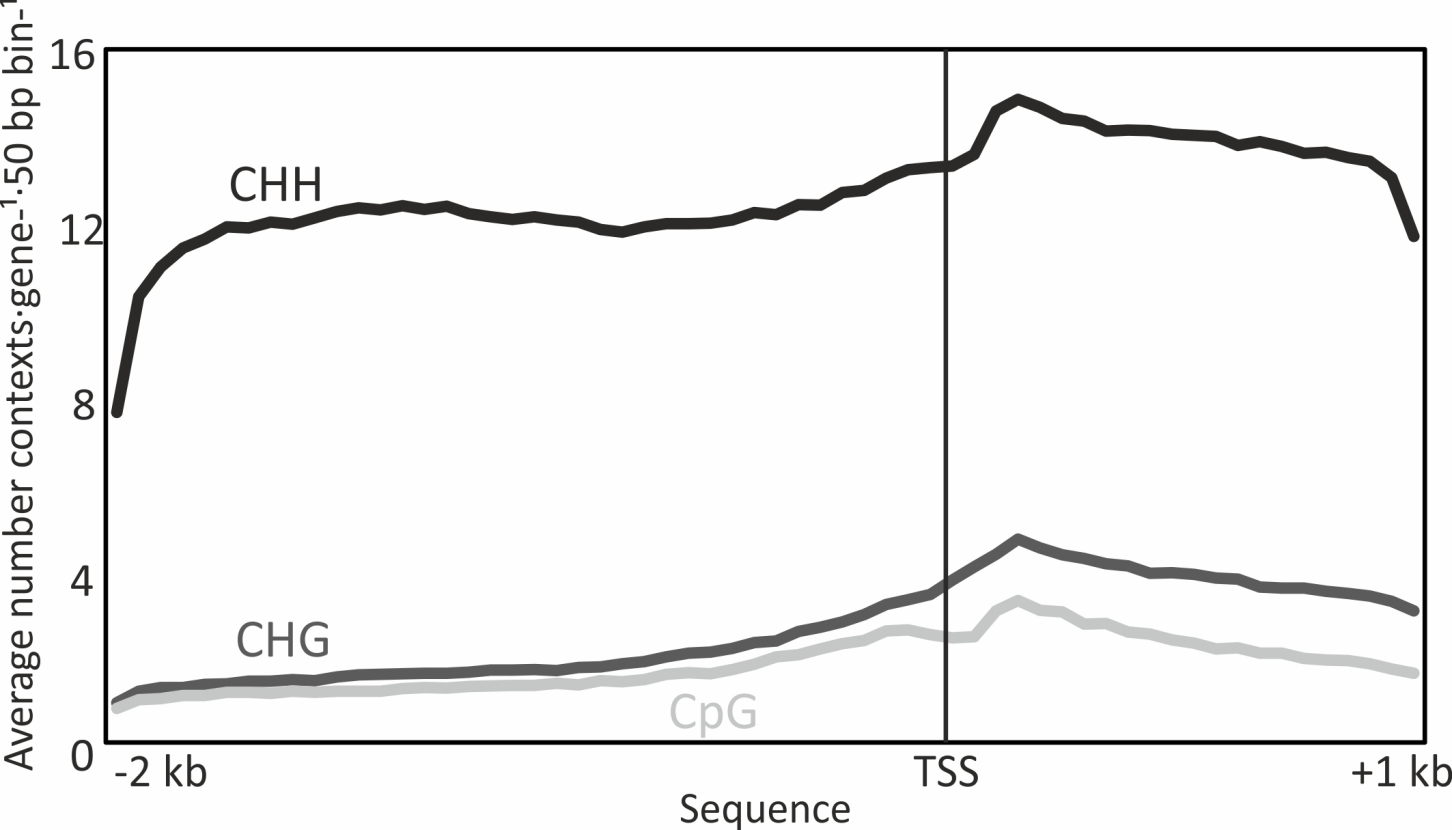
Abundance of CHH, CHG and CpG (where H = A, T or C) methylation sites irrespective of their DNA methylation status, in 50 bp windows along 2 kb of the promoter and 1 kb of the gene body spanning the transcriptional start site (TSS) of targeted Norway spruce genes. Data are means of 2744 genes in n = 6 biological replicates (each consisting of tissue from five pooled embryos).

Irrespective of their methylation status, the abundance of CHH, CHG, and CpG sites differed considerably along the analyzed 3 kb sequences of the target genes (**Figure 2**). The average prevalence per 50 bp window was, as expected, much higher for CHH sites than for CHG and CpG sites (there are many more possible base combinations for CHH sites). All three contexts showed a similar trend in prevalence along the analyzed 3 kb sequence, with a gradual increase towards a peak at the start of the gene body and lower abundance at the end of the studied gene body sequence.

**Figure 3:**
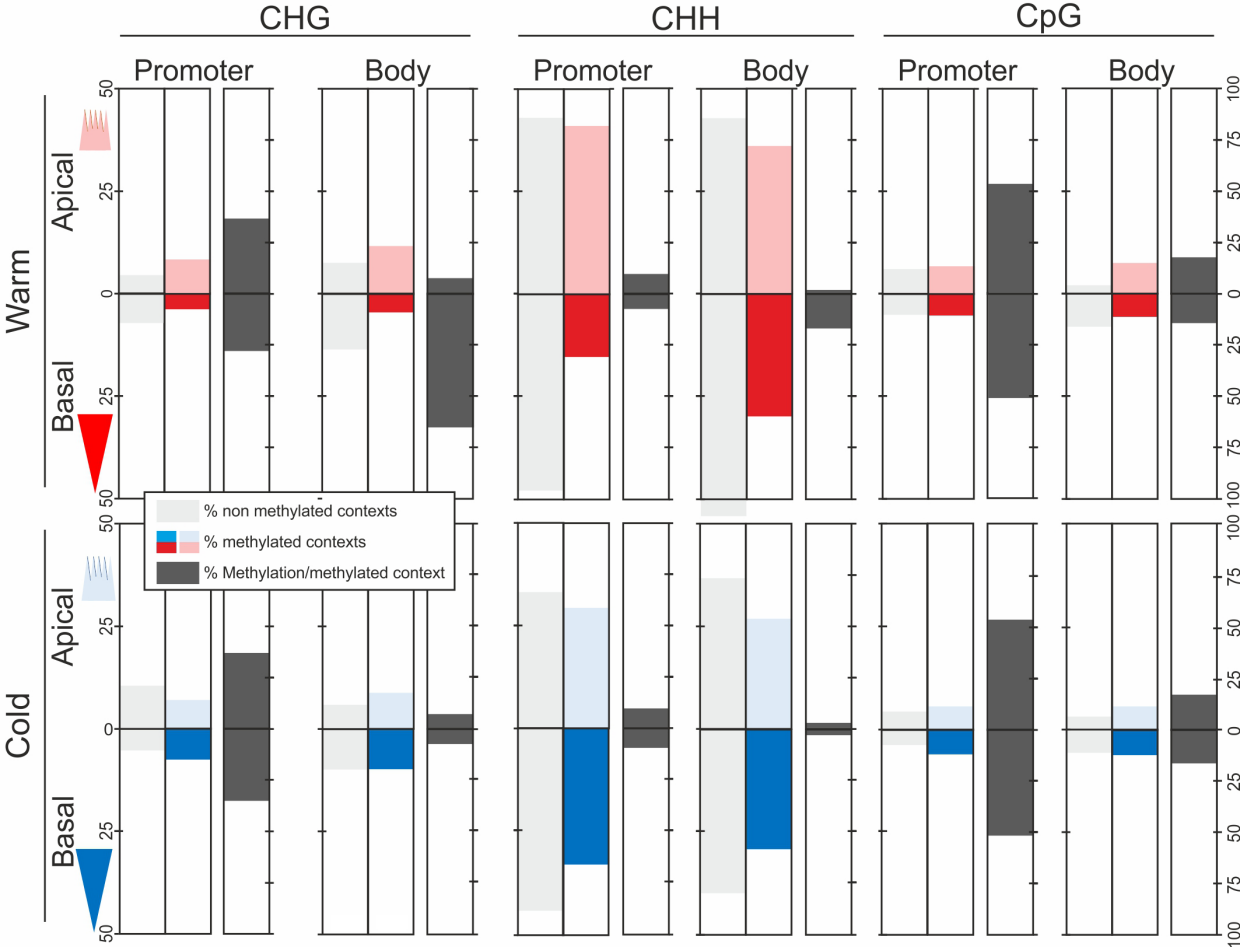
Unmethylated and methylated context sites and average percentage of DNA methylation per methylation context (CHG, CHH and CpG, where H = A, T or C) in Norway spruce. DNA methylation was determined across 2 kb of the promoter and 1 kb of the gene body spanning the transcriptional start site in 2744 genes. DNA was extracted from basal (darker blue/red) and apical parts (lighter blue/red) of genetically identical mature somatic embryos (i.e., epitypes) generated at warm (28°C; red) or cool (18°C; blue) temperatures. The sum of percentages of unmethylated or methylated context sites adds to 100% for each embryo part, epitype, and gene region (CHG + CHH + CpG = 100). Data are means of three biological replicates per embryo part and epitype, with each replicate consisting of pooled tissues from five embryos.

To determine the contribution of the three DNA methylation contexts to the methylation levels in each epitype, in apical and basal embryo parts, and in promoter and gene body regions we calculated the average percentages of unmethylated contexts (methylation level = 0), methylated contexts (methylation level > 0), and the average methylation level for each methylation context (**Figure 3**). CHH sites (i.e., all sites, methylated or not) over the 3 kB sequence were overrepresented compared to CHG and CpG sites. However, since very few CHH sites were methylated, their contribution to global methylation levels was very limited. On the other hand, there were few CpG sites, but due to their high methylation levels they contributed considerably to global methylation levels, mainly in the promoter region, regardless of epitype and embryo part. A similar pattern was observed for CHG sites, except that these had higher methylation levels per site in gene bodies in basal embryo parts in the warm epitype (**Figure 3**).

We also compared which methylation context sites that tended to be methylated or unmethylated between epitypes or embryo parts. CHG, CHH and CpG sites that were always unmethylated were shared between epitypes or embryo parts far more often than methylated sites, regardless of the methylation context and gene region (**Table 1**). The proportion of shared unmethylated sites ranged from 62-92%, whereas 20-44% of context sites with some level of methylation were shared. Overall, the CpG context had the highest percentages of both unmethylated and methylated sites, hinting at specificity in the mechanism targeting that context, followed by CHG and CHH. Gene bodies had fewer common methylated or unmethylated sites than promoters in all the comparisons, except for unmethylated CHH context sites, where the pattern was opposite.

**Table 1:** Percentages of commonly methylated and unmethylated DNA methylation contexts between epitypes in the apical and basal parts of genetically identical somatic embryos of Norway spruce developed under 18°C or 28°C.

		Common contexts (%)				
		Unmethylated			Methylated	
Comparison	Epitype	Promoter	Body		Promoter	Body
**Embryo parts** BASAL VS APICAL	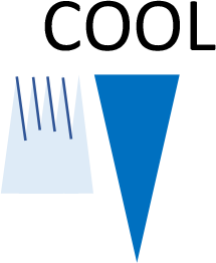	80	80	CHG	44	28
		63	72	CHH	29	26
		85	84	CpG	38	33
	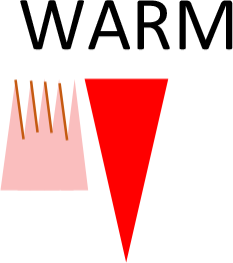	78	76	CHG	33	25
		65	69	CHH	30	20
		81	78	CpG	36	28
**EPITYPES** COOL VS WARM	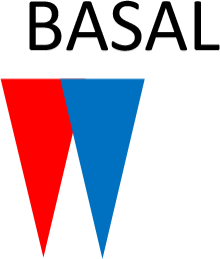	73	70	CHG	33	25
		62	65	CHH	28	20
		78	73	CpG	36	29
	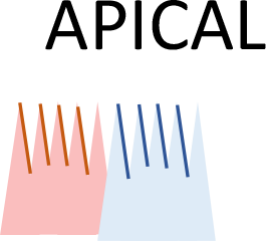	86	88	CHG	36	32
		62	75	CHH	30	28
		89	92	CpG	38	34

The percentages were calculated by dividing the total number of unmethylated and methylated contexts in the 2744 target genes by the total number of unmethylated and methylated contexts, respectively. The numbers are the means of three biological replicates, each consisting on 5 embryos per embryo part and epitype. Warm epitype is represented in red and cool epitype in blue; basal embryo parts are represented as inverted triangles (dark blue or red) and apical embryo parts as crown-like shapes (light blue or red).

### 3.3 Differentially methylated genes between epitypes

Most of the targeted genes in each functional category contained DMRs (with at least one differentially methylated 50 bp-bin) between epitypes in at least two of the three methylation contexts (**Table 1**). Genes that contained DMRs were classified as differentially methylated genes (DMGs).

**Figure 4:**
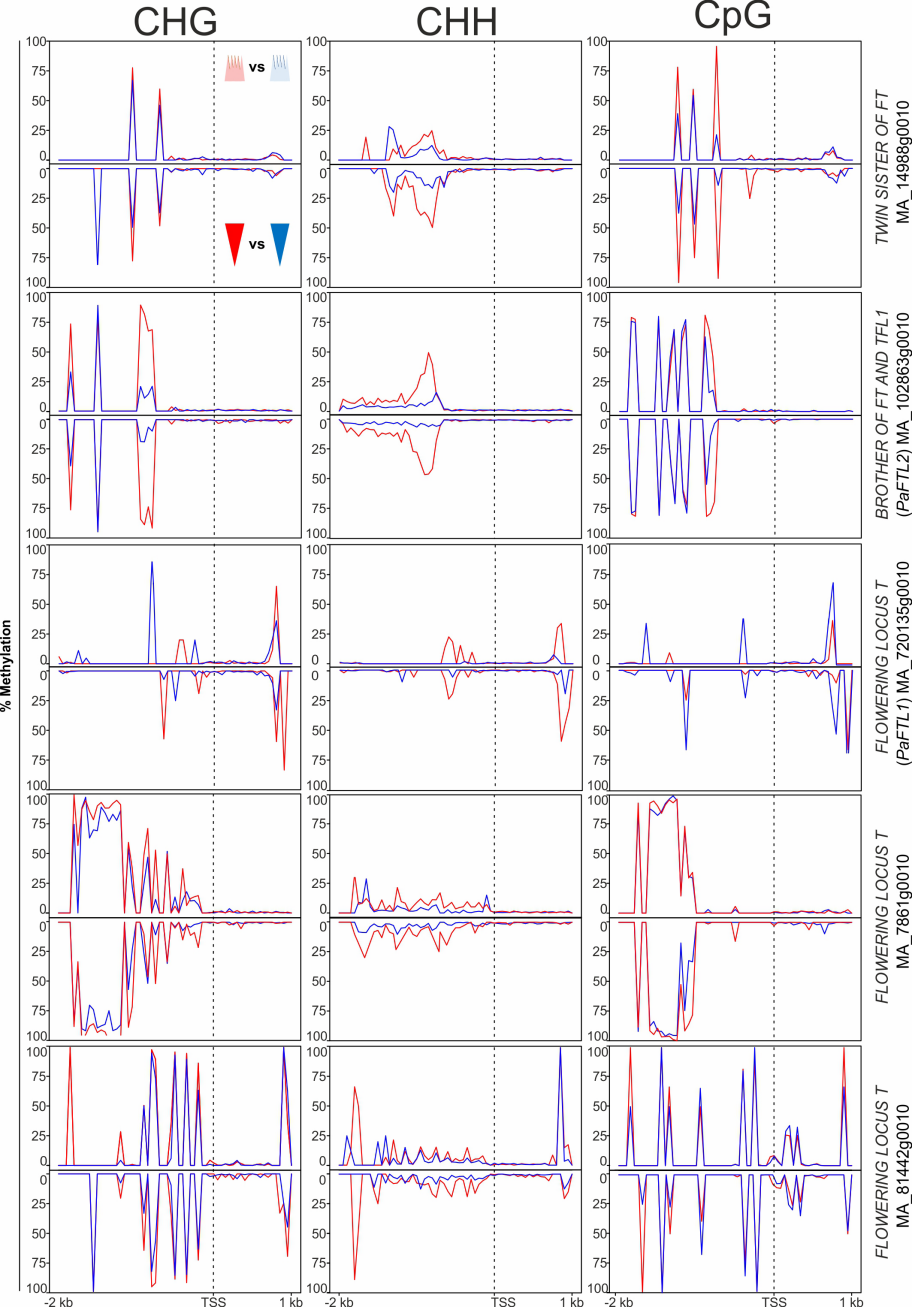
Average DNA methylation levels in 50 bp bins across 2 kb of the promoter and 1 kb of the gene body spanning the transcriptional start site (TSS; vertical, dotted line) of FT-TFL1-like genes in Norway spruce. Panels show methylation in apical embryo parts (crown-like shape; upper panel for each gene) and basal embryo parts (inverted triangle; lower panels) of genetically identical mature somatic epitype embryos generated at cool (18°C; blue) or warm (28°C; red) temperatures. Data are means of three biological replicates per embryo part and epitype, with pooled materials from five embryos per sample.

**Figure 5:**
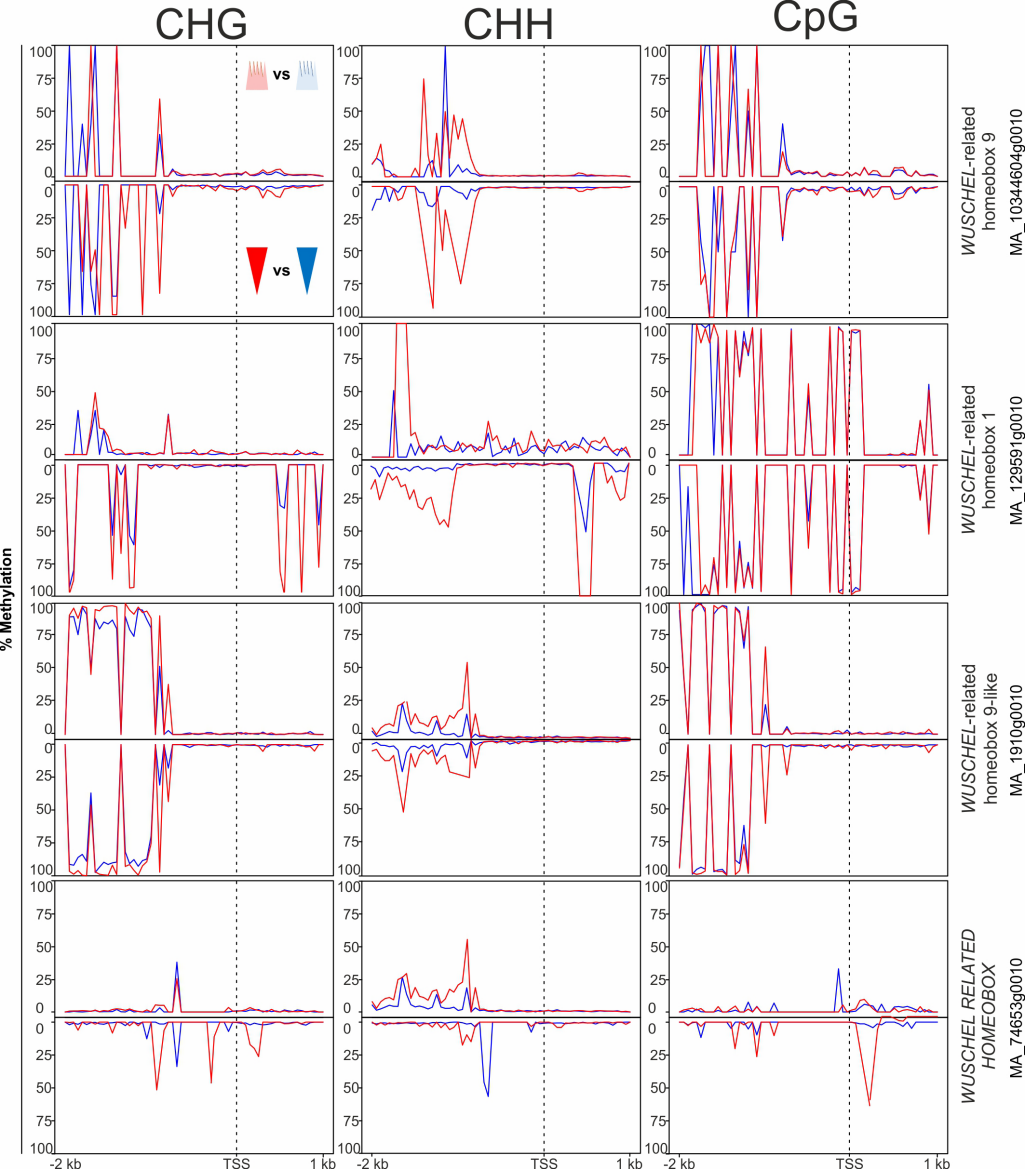
Average DNA methylation levels in 50 bp bins across 2 kb of the promoter and 1 kb of the gene body spanning the transcriptional start site (TSS; vertical, dotted line) of targeted WUSCHEL-like genes in Norway spruce. Panels show methylation in apical embryo parts (crown-like shape; upper panel for each gene) and basal embryo parts (inverted triangle; lower panels) of genetically identical mature somatic epitype embryos generated at cool (18°C; blue) or warm (28°C; red) temperatures. Data are means of three biological replicates per embryo part and epitype, with pooled materials from five embryos per sample.

In the ‘growth and development’ gene category, 18 genes potentially involved in phenological response were DMGs. These included genes associated with meristem identity [*WUSCHEL* (*WUS*) and *APETALA 2* (*AP2*)], embryo development [*LATE EMBRYOGENESIS ABUNDANT* (L*EA*)], light input to the circadian clock and its normal oscillation [*CONSTANS-LIKE* (*COL*) and *SENSITIVITY TO RED LIGHT REDUCED 1* (*SRR1*)], vernalization and the regulatory network controlling meristem determination, bud set, and photoperiodic control of flowering [MADS box gene *SOC1*, *FLOWERING LOCUS T-TERMINAL FLOWER 1-LIKE 1* and *2* (*PaFTL1, PaFTL2*), *FLOWERING LOCUS T-like* genes, *TWIN SISTER OF FT* (*TSF*) and *LATE FLOWERING* (*LF*) (some displayed in **Figure 4**)]. Seven of the eight homologs of the *FT-TFL1*-like genes in Norway spruce included in our study showed specific differential methylation patterns in all methylation contexts. These genes had more epitype-specific CpG DMRs in the basal embryo part than in the apical part (**Supplementary Table 2**; **Figure 4**). There was also high variability in the DMRs for the four WUSCHEL homologs analyzed, both with respect to methylation contexts and embryo parts (**Figure 5**).

**Figure 6:**
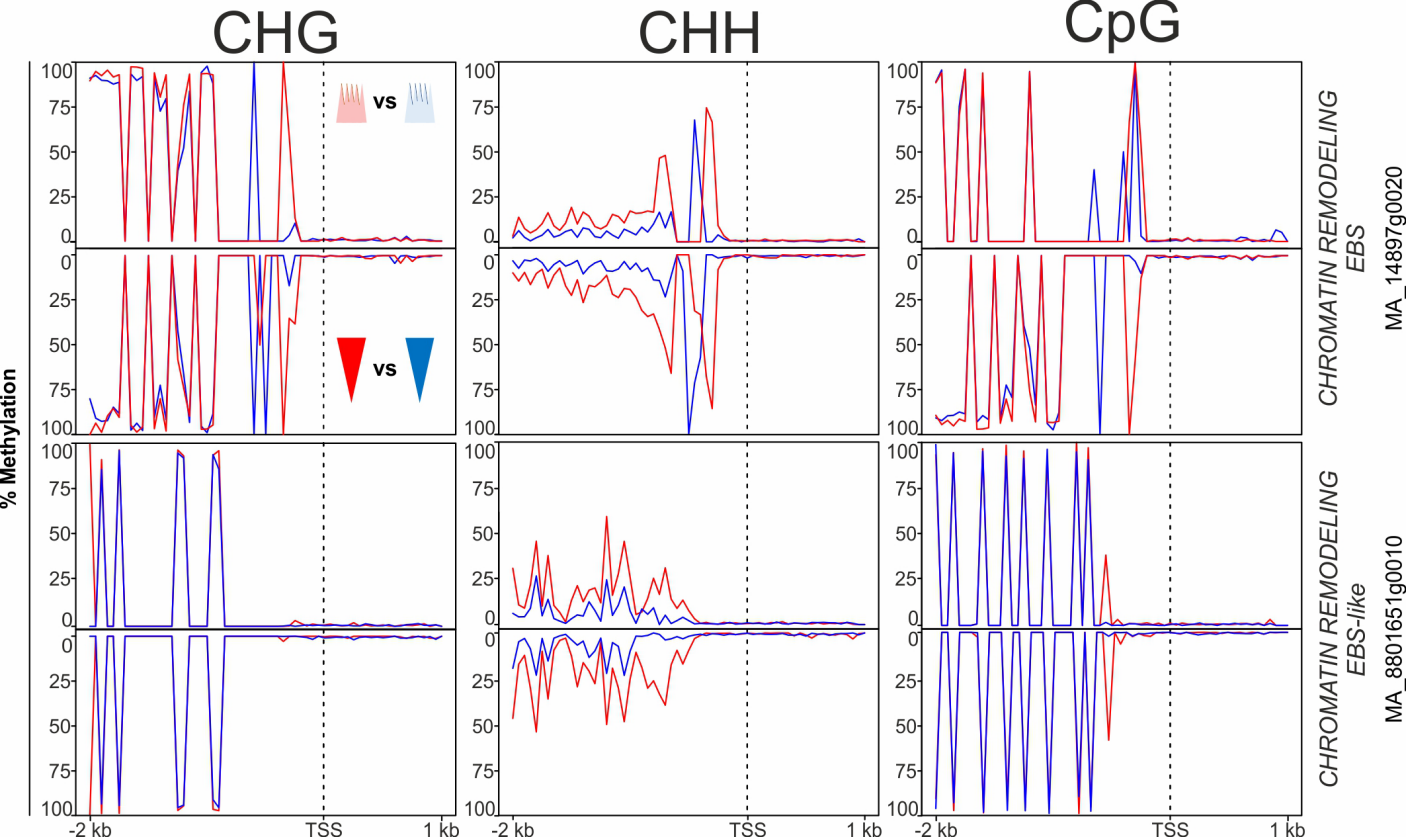
Average DNA methylation levels in 50 bp bins across 2 kb of the promoter and 1 kb of the gene body spanning the transcriptional start site (TSS; vertical, dotted line) of targeted chromatin remodeling genes in Norway spruce. Panels show methylation in apical embryo parts (crown-like shape; upper panel for each gene) and basal embryo parts (inverted triangle; lower panels) of genetically identical mature somatic epitype embryos generated at cool (18°C; blue) or warm (28°C; red) temperatures. Data are means of three biological replicates per embryo part and epitype, with pooled materials from five embryos per sample.

Furthermore, several genes in the epigenetic machinery category, putatively involved in the establishment of the epigenetic memory during embryogenesis, were DMGs (**Supplementary Table 2**). DMRs within such genes were most frequent in the CHH context, followed by CHG and CpG (**Supplementary Table 2**, ‘Epigenetic’ tab). The high number of paralogues involved in DNA methylation and miRNA generation (i.e., *ARGONAUTE*-like genes) that contained DMRs was noteworthy (**Supplementary Table 2**). Additionally, DMGs included several homologues to genes involved in the epigenetic control of flowering timing and the circadian clock. These genes encode chromatin remodelers that modulate the content of epigenetic marks in the chromatin (e.g., *EARLY BOLTING IN SHORT DAYS*, *CHROMATIN REMODELING PROTEIN EBS*-*like* and *CHROMATIN REMODELING PROTEIN EBS*; **Figure 6**). Most DMRs in genes in this category were hypermethylated in the warm epitype both in apical and basal embryo parts relative to their cool epitype counterparts (as exemplified in **Figure 6**).

### 3.4 Overview of differentially expressed genes during embryo maturation

**Figure 7:**
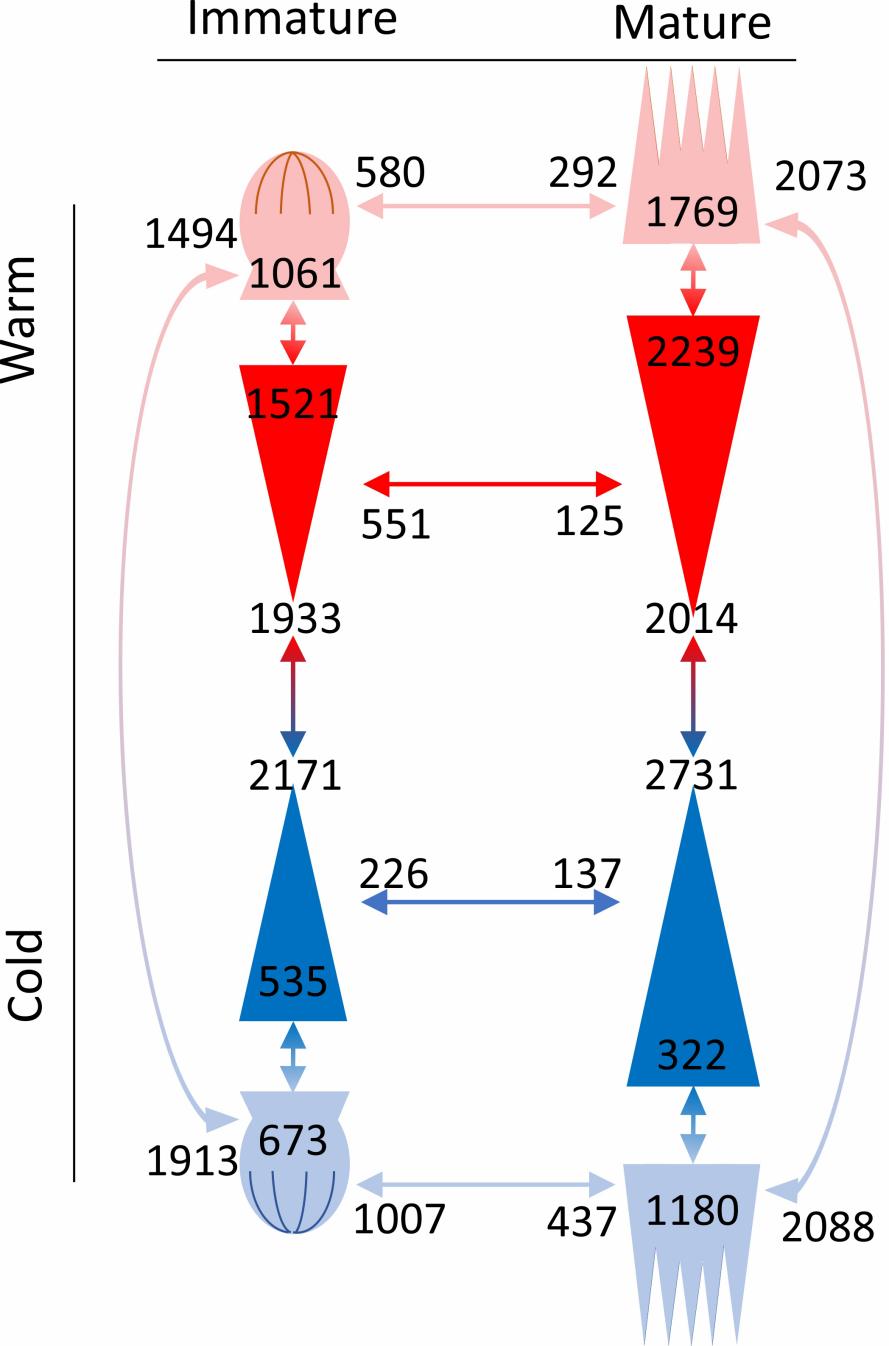
Number of differentially expressed genes in comparisons (arrows with associated numbers) between epitypes (red/blue arrows), embryo parts or developmental stages (red or blue arrows depending on epitype). Within each comparison, genes that are upregulated on one side of the arrow are downregulated on the other. Drawings represent the apical (crown-like shapes) and basal parts (triangles) of immature and mature developmental stages of genetically identical somatic epitype embryos of Norway spruce exposed to 18°C (cool; blue) or 28°C (warm; red) during embryogenesis. Note the increase in embryo size towards maturity. Data are means of three biological replicates per embryo part and epitype, with pooled materials from five embryos per replicate.

Generally, there were fewer DEGs between epitypes in the immature embryos than in the mature embryos (**Figure 7**). In addition, compared to the cool epitype, the warm epitype had more DEGs between embryo parts at each developmental stage. Also, the warm epitype had fewer upregulated genes in the apical part of the embryo compared to the basal part, whereas the cool epitype showed the opposite trend.

We analyzed the transcriptomic profiles of genes belonging to the epigenetic machinery in immature and mature embryos (**Supplementary Table 3**). We focused on the apical part of the embryos since it contains the shoot apical meristem that gives rise to above-ground plant parts such as buds, which are responsible for timing important phenological events, such as bud set and bud burst. Interestingly, we did not find any genes encoding DNA demethylation enzymes that were differentially expressed between embryo parts, epitypes or developmental stages. By contrast, genes encoding histone acetylation and deacetylation enzymes were differentially expressed in all the comparisons.

As the embryos matured there was a decreasing and very limited number (16) of DEGs related to the epigenetic machinery in the apical embryo part of each epitype. Twelve genes mainly involved in DNA methylation and sRNA processing and silencing were upregulated in mature vs. immature embryos of the cool epitype, while there was no upregulation in immature vs. mature embryos (**Supplementary Table 3**). Three genes related to histone methylation and acetylation were upregulated in mature vs. immature embryos of the warm epitype and one gene involved in sRNA processing was upregulated in immature vs. mature embryos.

In the apical part of immature epitype embryos, 50 epigenetically-related genes were upregulated in the warm vs. cool epitype, whereas 10 such genes were upregulated in the cool vs. warm epitype. In mature embryos, 46 and 27 genes were upregulated in the same embryo part and epitypes (**Supplementary Table 3**).

## 4 Discussion

### 4.1 Epitypes differ greatly in DNA methylation marks

Different epi-temperatures are known to induce an epigenetic memory in somatic as well as zygotic embryos in Norway spruce, resulting in epigenetically different plants (epitypes) with distinct bud phenology and differential adaptation to local climatic conditions (Kvaalen and Johnsen, 2008; Yakovlev and Fossdal, 2017). By using targeted bisulfite sequencing, we demonstrate that contrasting cool and warm epi-temperatures during somatic embryogenesis in Norway spruce induce consistent and different DNA methylation patterns in the resulting embryo epitypes. Targeted, high resolution cytosine methylation analysis (Wendt et al., 2018) of 2 kb of the promoter and 1 kb of the gene body spanning the TSS of 2744 target gene regions revealed significant differences between epitypes in genes regulating phenology, the epigenetic machinery, and the circadian clock. Methylation and gene expression analyses of apical and basal embryo revealed that the epi-temperature induce substantial differences also between these embryo parts, containing the shoot and root apical meristem, respectively.

DNA methylation in plants is complex, both functionally and because three different methylation contexts are involved (reviewed in Neiderhuth and Schmitz, 2014). Based on our results we conclude that embryo development under different epi-temperatures in Norway spruce induces the formation of unique and reproducible temperature-associated DNA methylation fingerprints. This conclusion is supported by the considerable methylation difference observed between embryo epitypes, with low percentages (20-38%) of shared methylation across the three methylation contexts and relatively high percentages (65-92%) of shared unmethylated contexts (**Table 1**). This high number of unique methylated contexts between epitypes is consistent with the phenotypic differences between the epitype plants that develop from these epigenetically different embryos.

The analyzed promoter regions tended to have more DNA methylation than the coding part in the gene bodies. Although the contrasting mature embryo epitypes had different average DNA methylation levels along the investigated 3 kb sequences, the overall methylation patterns shape were similar between the two epitypes: a progressive reduction in DNA methylation along the 2 kb promoter sequence towards the TSS, and a low and rather constant DNA methylation level along the first 1000 bp of the gene body (**Figure 2**). Also, average methylation frequencies in the CHG, CpG and CHH contexts in our epitype embryos were similar to those observed for gymnosperms in previous studies (Ausin et al., 2016; Takuno et al., 2016; Heer et al., 2018). However, in contrast to chickpea (Rajkumar et al., 2020), shoot stem cells of *A. thaliana* (Gutzat et al., 2020) and Norway spruce needles (Heer et al., 2018), we did not observe any sharp drop in DNA methylation at the start of the gene body (**Figure 2**).

Previous investigations of the epigenetic memory in Norway spruce embryos used entire embryos and studied transcriptomes and small non-coding RNAs, not DNA methylation (Yakovlev et al., 2016; Yakovlev and Fossdal, 2017). In this study we have, for the first time, analyzed methylation patterns in specific genes in different embryo parts containing the shoot and root apical meristem. These analyses revealed striking differences at the DNA methylation level: ratios of methylated and unmethylated CHG, CpG and CHH contexts varied between epitypes and between apical and basal embryo parts, across both promoter and gene body regions of several target genes (**Figure 4**). Also, different DNA methylation contexts had different spatial distributions of methylated and unmethylated sites. Notably, CHG contexts in the first 1 kb of the gene body were highly hypermethylated in the basal part of the warm epitype relative to the cool epitype (**Figure 4**), showing that different temperatures induce differential DNA methylation patterns. These findings support the hypothesis that the epigenetic landscape of Norway spruce epitypes is shaped by the temperature conditions experienced during embryo development (Johnsen et al., 2005; Yakovlev et al., 2016). Furthermore, although the exact role of gene body-methylation has been described as enigmatic and its exact functional role has been questioned (Bewick and Schmitz, 2017; Bewick et al., 2017), our findings point to a fine-tuning of methylation and suggest a potential biological role of CHG gene body methylation in epigenetic memory.

### 4.2 Differential gene methylation may define the epigenetic memory

Phenological traits are important for the survival of long-lived species such as Norway spruce. Epitype trees grown from cool and warm epitype embryos show adaptive differences in phenology (Kvaalen and Johnsen, 2008; Carneros et al., 2017), and we have previously hypothesized that epitypes differ in DNA methylation of genes involved in timing of autumnal bud set and spring bud burst. Previously, increased expression of *FT*-*TFL1 (FTL)*-like genes has been shown to correlate with short-day (SD)-induced winter bud formation in Norway spruce, whereas reduced expression of *FTL-like* genes correlate with bud break (Gyllenstrand et al., 2007; Asante et al., 2011). Also, a causal relationship between expression of *FTL2* and SD-induced bud set was established using *FTL2*-overexpressing plants (Karlgren et al., 2011). *FTL* genes belong to the phosphatidylethanolamine-binding (PEBP) gene family (Karlgren et al., 2011), and in our study several *FTL*-like genes in Norway spruce had epitype-specific DNA methylation patterns (**Figure 6**). This may suggest that DNA methylation of these genes play a role in the epigenetic memory effect. Furthermore, a previous study of epitype trees originating from somatic embryos generated at 18 and 28°C showed significantly different *FTL2* expression between epitypes in terminal buds prior to and during bud burst (Carneros et al., 2017). This, together with the gene-specific DNA methylation patterns described here for *FTL* genes, suggest that genes controlling phenological traits are involved in the epigenetic memory of Norway spruce.

We also found that effects of epi-temperatures on DNA methylation patterns extended to genes such as the MADS-box gene *SOC1*, whose expression decreases under conditions leading to bud set in Norway spruce (Opseth et al., 2016; Chiang et al., 2021). However, because differences in DNA methylation between embryo epitypes were not unequivocally correlated with differential gene expression in our study, we cannot establish a firm connection between DNA methylation patterns and differential gene expression. Only two of the seven differentially methylated *FTL*-genes were differentially expressed in mature embryos [*BROTHER OF FT AND TFL1* (*PaTFL1*) and *TWIN SISTER OF FT*; **Figure 6**]. This may suggest that differences in DNA methylation between epitype embryos are due to changes in the epigenetic machinery that are induced earlier during embryogenesis. Thus, any effect these methylation marks have on the transcription of *FTL*-like genes may not manifest itself in the plants before buds are formed. We also observed differential methylation between epitypes in genes related to output pathways of the circadian clock function, such as the *SSR1*-like gene (sensitivity to red light reduced) (Johansson and Staiger, 2014) and *CONSTANS-LIKE 1* (*COL1*) (Holefors et al., 2009). These differences suggest that the epigenetic status of circadian clock-associated genes in Norway spruce may be fixated during embryogenesis and that this later affects the regulation of bud phenology. Moreover, the fact that all but one of the many *FTL*-like genes downstream from the circadian clock-related genes were differentially methylated between epitypes or embryo parts supports the hypothesis that *FTL*-like genes are involved in epigenetic memory formation in Norway spruce.

We also observed differential methylation patterns in key meristem-related genes during somatic embryogenesis. These genes include *WUSCHEL*-like (**Figure 7**) and *APETALA2*-like genes that are involved in meristem maintenance and determination (Würschum et al., 2006), as well as *APETALA2* that is important for embryo development (Ohto et al., 2009). There is increasing evidence for epigenetic regulation of *WUSCHEL*, a gene important for maintaining stem cell pools in shoot apical meristems and floral meristems, as well as *de novo* organogenesis (Mayer et al., 1998; Cao et al., 2015); Zhao et al., 2008; Cheng et al., 2014). Also, in *A. thaliana* expression of *APETALA3* is impacted by methylation in its promoter (Wang et al., 2019). Our results for *WUSCHEL*-like and *APETALA2*-like Norway spruce gene models are consistent with earlier studies and suggest that DNA methylation is involved in controlling gene expression in shoot and root apical meristems.

### 4.3 The epigenetic memory is associated with differential DNA methylation and expression of epigenetics-related genes

Previous studies have shown that DNA methylation and posttranslational histone modifications work together to establish open or closed chromatin states (Soppe et al., 2002; Jasencakova et al., 2003; Ebbs and Bender, 2006; Liu et al., 2012). Our results are consistent with this general model. First, more than 50% of all genes related to the epigenetic machinery had differential DNA methylation patterns between mature embryo epitypes. Second, different epi-temperatures had a major impact on DNA methyltransferases and genes involved in posttranslational histone modifications (e.g., histone lysine N-methyltransferases, histone acetyltransferases, and histone deacetylases).

Chromatin remodeling involving *EARLY BOLTING IN SHORT DAYS* (*EBS*)-like genes is an example of epigenetic control of genes in the photoperiodic pathway (Källman et al., 2014). In plants, EBS acts through histone 3-acetylation to regulate genes in the photoperiod pathway, such as *SOC* and *FT* (López-González et al., 2014). We found two EBS-like genes with different DNA methylation levels between embryo epitypes (**Figure 6**). Because these genes also were differentially expressed during embryo development (**Supplementary Table 3**) they are interesting candidates for further studies of the epigenetic memory of Norway spruce.

Genes involved in DNA methylation were overrepresented among the upregulated genes in the warm epitype relative to the cool origin epitype. In contrast, there was no differential expression related to DNA demethylation. This is noteworthy, since this correlates with the higher levels of DNA methylation detected in mature embryos of the warm epitypes. This increased transcript level of DNA methylation genes during embryogenesis could be the cause of the higher DNA methylation levels observed in the mature embryos exposed to warmer conditions. Whether the DNA methylation patterns we observe in embryos are retained also in the resulting trees and how these epigenetic marks are related to the epigenetically altered bud phenology will be a priority for future research.

Some classes of small RNAs (sRNAs) are known to be important in epigenetic processes, such as interfering RNAs that drive RNA-directed DNA methylation (RdDM) (Miguel, 2020). We observed epitype-specific differences in DNA methylation of genes involved in sRNA biogenesis and sRNA-mediated signaling, such as *ARGONAUTE* (**Supplementary Table 2**). These observations are consistent with previous results by Yakovlev et al. (2016), who showed highly dynamic sRNA reprogramming in Norway spruce during somatic embryogenesis at different epi-temperatures. Interestingly, novel sRNAs, including a 31 nt type not previously described in plants, have recently been identified in Norway spruce embryos together with numerous miRNA families and other sRNAs yet to be characterized. Due to their differential expression during epitype-inducing temperature conditions these sRNAs have been proposed to be effectors involved in the formation of the epigenetic memory (Yakovlev and Fossdal, 2017).

This is the first report in any plant species of a temperature-induced epigenetic memory causing differential DNA methylation patterns in embryos. Importantly, different epi-temperatures induced differential methylation between epitypes in most of the studied genes putatively involved in DNA methylation, histone modifications, small RNA processing, and bud phenology. This indicates that the DNA methylation marks we observed may be part of an induced epigenetic memory that is later manifested as phenologically different epitype trees. The observation that also genes related to histone modifications (39 genes) were differentially methylated indicates that this epigenetic mechanism could be important in the epigenetic memory of Norway spruce.

## Data availability statement

The datasets presented in this study can be found in online repositories. The names of the repository/repositories and accession number(s) can be found below: https://www.ncbi.nlm.nih.gov/, BioProject PRJNA939017.

## Author contributions

MV conceived the experimental design, generated and processed the plant tissues, interpreted the generated data and wrote the article. CF and IY conceived the experimental design, interpreted the data and wrote the article. TT did the bioinformatic analyses and partially wrote the associated parts in material and methods and results. PK did significant revision of the manuscript contents. HC did the bioinformatics associated with the experimental design. JO conceived the experimental design, interpreted the results and did significant revision of the manuscript. All authors contributed to the article and approved the submitted version.
